# The management of severe hypertension in Australian general practice

**DOI:** 10.1186/1472-6963-13-414

**Published:** 2013-10-14

**Authors:** Blanca Gallego, William B Runciman, Oscar Perez-Concha, Siaw-Teng Liaw, Ric O Day, Adam G Dunn, Enrico Coiera

**Affiliations:** 1 Centre for Health Informatics, Australian Institute of Health Innovation, University of New South Wales, Sydney 2052, Australia; 2 School of Psychology, Social Work and Social Policy, University of South Australia, Adelaide SA 5000, Australia; 3 School of Public Health and Community Medicine, University of New South Wales, Sydney 2052, Australia; 4 St Vincent’s Clinical School, University of New South Wales, St Vincent’s Hospital, Darlinghurst NSW 2010, Australia

**Keywords:** Severe hypertension, Electronic general practice records, Chronic disease management

## Abstract

**Background:**

Severe hypertension (SHT) (Blood Pressure, BP ≥ 180/110 mmHg) is associated with considerable morbidity and mortality, yet little is known about how it is managed. The purpose of this study is to examine the management of SHT by Australian general practitioners (GPs) and to explore its variance across patient characteristics and clinical practices.

**Methods:**

Review of electronic medical records for a year before and after a recorded measure of SHT in 7,499 patients by 436 GPs in 167 clinics throughout Australia during 2008–2009. Outcome measures included follow-up, referral, changes to antihypertensive drug treatment, and BP control (normotensive reading, BP < 140/90 mmHg, and whether subsequent recorded measures were also in the normal range - sustained normotension).

**Results:**

Of 7,499 patients with an electronic BP record of SHT, 94% were followed up (median time 14 days); 8% were referred to an appropriate specialist (median time 89 days – 2% within 7 days) and 86% were managed by GPs. GPs initiated or changed antihypertensive drugs in 5,398 patients (72% of cohort); of these, 46% remained hypertensive (4% with SHT) and 7% achieved sustained normotension; 6% had no further electronic BP records. The remaining 14% had no medication changes; among these, 43% remained hypertensive (5% with SHT) and 3% achieved sustained normotension; 32% had no further electronic BP records. Some outcome measures displayed a variance across GP clinics that was mostly unexplained by patient or practice characteristics.

**Conclusions:**

Most patients with SHT had at least one follow-up visit and 72% had initiation of, or changes to, antihypertensive drug treatment. Although most of the patients experienced some improvement, blood pressure control was poor. Some clinics showed better performance. Suggestions are made for the development of clinical standards to facilitate appropriate management of this dangerous condition.

## Background

Hypertension is the most common primary diagnosis in general practice, affecting approximately 1 in 3 adults [1]. It is also the leading risk factor for premature mortality in the world, contributing to about 45% of all cardiovascular deaths [1-3], and is the second highest ranked cause of disability. There is compelling evidence that systolic blood pressure (BP) above optimal levels proportionally increases the risk of stroke, coronary heart disease and chronic kidney disease. In particular, individuals with severe hypertension (SHT), defined as systolic BP ≥ 180 mmHg or diastolic BP ≥ 110 mmHg, are at serious short-term risk of cardiovascular events, have 10 year cardiovascular disease risks of about 20%, and high rates of target organ complications [4-7].

Although hypertension is easy to diagnose and inexpensive to treat, a significant gap has been documented between recommended and received care [8-14]. A report by the Institute of Medicine in the United States calls hypertension a “neglected disease”, and attributed this to lack of adherence to treatment guidelines, since 86% of people with hypertension visit their doctors [15]. Other authors suggest that primary care physicians may not be aggressive enough in managing hypertension and are willing to accept an elevated systolic BP in their patients [16] - particularly in the elderly [17,18]. At the other end of the adult age band, authors of a large Australian study found that lack of treatment for hypertension was also associated with perceived low risk in young, non-obese, non-diabetic males [12].

Because of the dangers associated with SHT, prompt, adequate management is essential. The need for prompt follow-up and/or referral to a specialist, and appropriate combinations of drugs to achieve BP control (BP < 140/90 mmHg) is emphasized in guidelines [4,19]. Yet previous studies of SHT management [20-22] have provided evidence that treatment is often inadequate in relation to the serious risks faced by these patients.

In this paper we examine key aspects of the management of patients with SHT in Australian general practice. In particular, we focus on the responses by general practitioners (GPs) to the presence of SHT via follow-up, referral, and addition of, or changes to, antihypertensive drug treatment; and report on subsequent recorded BP measures during one year of follow-up. In addition we explore how much of the variance in follow-up and BP control can be explained by patient, doctor and practice characteristics.

## Methods

### Data

The electronic medical records analysed in this study were collected by the General Practice Research Network (GPRN) (http://www.hcn.com.au/Products/Strategic+Solutions) [23], a national network of Australian GPs. To date, more than 1,000 GPs have contributed to this dataset; in the study period under consideration there were 436 GPs in 167 clinics distributed across all 8 states and territories with a mean representation of 38 GPs per 100,000 inhabitants and 68% of these practices in metropolitan areas.

All patients with a BP measurement of SHT (BP ≥ 180/110 mmHg) between March 1, 2008 and March 1, 2009 were selected. Their records one year before and after the first recording of this SHT measure (reference visit) were examined. The 45 patients who died in the follow-up year were excluded since they represent a very small percentage of the sample size (<1%) and may have complications or other adverse events (not controlled for) other than hypertension. If more than one BP was recorded or there was more than one visit on a day, the BP values for that day were averaged.

The patient characteristics included in this study were sex, age (at reference visit), number of visits and recorded measures of SHT during the previous year, diabetes status, and the last available measure of total cholesterol to high density lipoprotein ratio (CHOL:HDL) up to and including the reference visit. Only 0.09% of patients had missing age; cholesterol measures were classified as >4.5, ≤4.5 or missing. The characteristics of GPs and practices included age and years from graduation (at March 2008), sex, country of graduation, practice size (the number of GPs in that practice – part-timers were weighted by 0.5) and practice location (rural or metropolitan). A very small number of GPs had missing age (1%), year of graduation (1%) and country of graduation (5%); and 1% of clinics had missing size.

Outcome measures were: (a) Follow-up visits to the same practice and time to first follow-up visit from the reference visit; (b) Addition of, or changes to, anti-hypertensive drug treatment during the year after the reference visit; these drugs were classified into angiotensin 1 (AT1) receptor blockers, angiotensin converting enzyme (ACE) inhibitors, beta-blockers, calcium channel blockers, diuretics and others; (c) first normotensive reading (BP <140/90 mmHg) (normotension), interval before first normotensive reading and whether all subsequent recorded measures were also in the normal range (sustained normotension). Outcome measures (b) and (c) were computed only for patients with at least one follow-up visit who were not referred to a specialist. Here, specialists included cardiology, endocrinology, renal medicine and/or geriatrics. Sustained normotension required a minimum of 2 BP measures.This study was approved by the UNSW Human Research Ethics Committee (09154).

### Statistical analysis

A hierarchical logistic regression model, with a patient and a GP clinic level, allowed us to investigate if the occurrence of a follow-up visit clustered by GP clinic and how much of the variance could be explained by some patient, GP and GP clinic characteristics. Failure to adhere or sustain normotension was also modeled using similar two-level hierarchical models. Practices with less than 20 SHT patients, as well as patients for whom normotension (or sustained normotension) could not be calculated, were excluded from the regression analysis. No imputation on other missing data (which was negligible) was performed.

Multilevel models recognise the existence of data hierarchies and are a preferred method for examining variations in outcomes across institutions, providers, and other relevant groups [24]. The parameters of the regression models were estimated using SAS PROC GLIMMIX with random intercepts, residual pseudo-likelihood estimation and the Cholesky algorithm [25]. We also computed the intraclass correlation coefficient (ICC), which described how strongly outcomes within one GP/practice resembled each other, and was estimated following the linear threshold method by Snijders and Bosker [26]; and the median value of the odds ratios’ second level (MOR) [27], which measured the extent to which an outcome was determined by the GP clinic.

## Results

**Figure 1:**
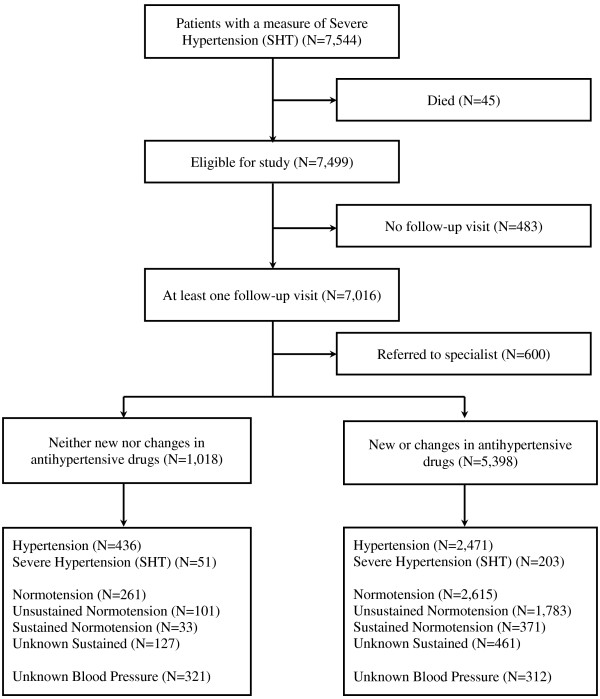
Summary illustration of the numbers of patients with severe hypertension (SHT) with respect to follow-up, referral, changes in antihypertensive drugs treatment and blood pressure (BP) control during the year of follow-up.

The selection criteria resulted in a sample of 7,499 patients with at least one recorded measure of SHT (see Figure 1); these had 78,757 visits to 436 GPs in 167 GP clinics during the follow-up year (including the reference visit). The variables characterising these patients, GPs and GP clinics are summarised in Table 1.

**Table 1:** Summary statistics

**Patients (n=7,499) mean number of visits per patient in the following year = 11**	
Sex	Male = 41%
	Female = 59%
Age	Median 68 years (25th percentile 55 years, 75th percentile 78 years)
Visits during previous year	Median 2 visits (25th percentile 0 visits, 75th percentile 5 visits)
Severe hypertension measures during previous year	Median 0 measures (25th percentile 0 measures, 75th percentile 0 measures)
Total cholesterol to HDL ratio	Unknown = 73%
	≤ 4.5 = 7%
	>4.5 = 20%
**General practitioners (n=436) mean number of visits per general practitioner in following year = 181**	
Sex	Male = 65%
	Female = 35%
Age	Median 48 years (25th percentile 39 years, 75th percentile 56 years)
Years from graduation	Median 23 years (25th percentile 15 years, 75th percentile 31 years)
**General practice clinics (n=167) mean number of visits per clinic in following year = 472**	
Number of general practitioners in clinic*	Median 2 GPs (25th percentile 1 GP, 75th percentile 4 GPs)
Practice location	Metropolitan = 68%
	Rural = 32%

*Weighted as 0.5 if doctor works.

**Figure 2:**
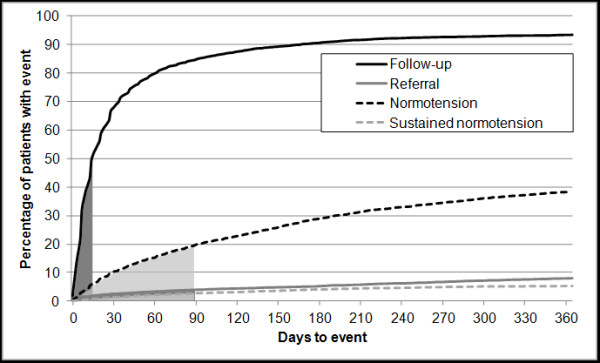
**Times to follow-up, referral, a normotensive reading and sustained normotension during the study year.** Percentages are calculated with respect to the full cohort of 7,499 patients. Shaded areas correspond to 50% of patients (median time).

Most of the 7,499 patients (94%) had a follow-up visit with a median of 14 days (see Figure 2); 66% of these patients visited more than one doctor within the same practice. Referral to an appropriate specialist took place in 8% of patients after a median of 89 days – 2% within 7 days (see Figure 2). The remaining 6,416 patients (86%) were managed solely by GPs. Older, diabetic patients or patients with previous SHT episodes were more likely to be referred to specialists.

Antihypertensive drug treatment and BP control were analysed for the patients managed by their GPs; 14% (of the total cohort) had no change or addition to their antihypertensive drugs, 26% were prescribed one antihypertensive drug, 26% a combination of 2, and 20% a combination of three or more. Overall, 38% of patients had at least one normotensive reading after a median of 88 days, (see Figure 2). For 633 patients there was no electronic record of a further blood pressure measurement; the free narratives (possibly containing BP records) were not available for study.

Comparisons between patients with and without changes in antihypertensive medication are summarised in Table 2. Patients with new or changes in antihypertensive drug treatment were more likely to have a recorded BP measure during the study period (94% vs. 68%). They were also more likely to reach normotension (48% vs. 26%) and less likely to have further measures of SHT (4% vs. 5%).

**Table 2:** Summary statistics of blood pressure (BP) control

**Total**			**New or changes in antihypertensive drugs**				**p-value (χ2 test)**
			**No**		**Yes**		
**Sample size**	**6,416**	**(100%)**	**1,018**	**(100%)**	**5,398**	**(100%)**	
**Hypertension**	**2,907**	**(45.3%)**	**436**	**(42.8%)**	**2,471**	**(45.8%)**	**0.08**
Severe (SHT)	254	(4.0%)	51	(5.0%)	203	(3.8%)	0.06
**Normotension**	**2,876**	**(44.8%)**	**261**	**(25.6%)**	**(25.6%) 2,615**	**(48.4%)**	**<0.0001**
Unsustained	1,884	(29.3%)	101	(9.9%)	1,783	(33.0%)	<0.0001
Sustained	404	(6.3%)	33	(3.2%)	371	(6.9%)	<0.0001
Unknown	588	(9.2%)	127	(12.5%)	461	(8.5%)	<0.0001
**Unknown BP**	**633**	**(9.9%)**	**321**	**(31.6%)**	**312**	**(5.8%)**	**<0.0001**

### Variance in the occurrence of a follow-up visit

**Figure 3:**
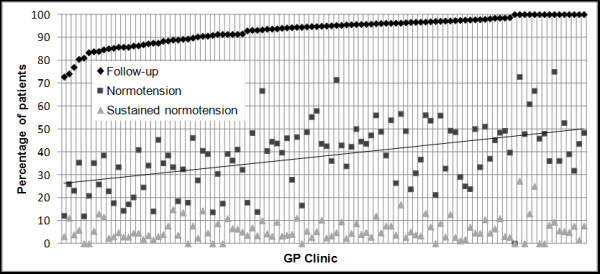
**Distribution of the percentage of patients with follow-up visits, who had a normotensive reading, and who achieved and sustained normotension by general practice (GP) clinic.** Only GP clinics with 20 or more severe hypertension (SHT) patients are included. Percentages are calculated with respect to the full cohort of 7,499 patients. Clinics were sorted from lowest to highest percentage value of follow-up visits.

Among the 106 clinics with at least 20 SHT patients, 14% provided follow-up visits for all their patients; the practice with the lowest follow-up had a rate of 73% (see Figure 3). There was a statistically significant difference in follow-up rates across GP clinics (p_*Fisher*_ < 0.0001); the median was 95% and the standard deviation was 6%; a similar variance was found across GPs.

All the patient characteristics had an independent, statistically significant association with lack of follow-up visit (see Table 3). Male, younger, non diabetic patients with unknown CHOL:HDL, fewer visits and fewer SHT recorded measures in the previous year were less likely to have a follow-up visit. For example, the odds ratio of having a follow-up visit if cholesterol measures were known was 3.4 [95% CI = (1.9, 6.1)]. Neither GP nor GP clinic characteristics had a statistically significant effect.

**Table 3:** Univariate and multivariate models of follow-up and blood pressure (BP) control

		Modelling lack of follow-up visit (106 clinics; 6,993 patients)*				Modelling hypertension (82 clinics; 5,057 patients)**				Modelling unsustained normotension (75 clinics; 4,431 patients)***			
		**Univariate analyses**		**Multivariate hierarchical regression**		**Univariate analyses**		**Multivariate hierarchical regression**		**Univariate analyses**		**Multivariate hierarchical regression**	
**Effect**		**Statistic†**	**(p-value)**	**Point estimate**	**(p-value)**	**Statistic†**	**(p-value)**	**Point estimate**	**(p-value)**	**Statistic†**	**(p-value)**	**Point estimate**	**(p-value)**
**Patient**													
Sex (ref=female)	male	χ^2^= 7.21	(0.03)	0.11	(0.3)	χ^2^= 33.37	(<0.0001)	0.33	(<0.0001)	χ^2^= 0.78	(0.68)	N/A	
Age		-0.03	(<0.0001)	-0.03	(<0.0001)	-0.008	(<0.0001)	-0.007	(0.0007)	0.01	(0.003)	0.01	(0.02)
Previous visits		-0.27	(<0.0001)	collinear with prev. SHT		-0.07	(<0.0001)	collinear with prev. SHT		-0.03	(0.01)	collinear with prev. SHT	
Previous SHT		-0.66	(<0.0001)	-0.45	(0.0008)	-0.09	(<0.0009)	0.17	(<0.0001)	-0.21	(0.02)	0.16	(0.07)
Total CHOL/HDL (ref=unknown)	>4.5	χ^2^= 69.70	(<0.0001)	-1.23	(<0.0001)	χ^2^= 30.16	(<0.0001)	-0.05	(0.64)	χ^2^= 0.20	(0.9)	N/A	
	≤4.5			-1.05	(<0.0001)			-0.32	(<0.0001)			N/A	
Diabetic Status (ref=no)	yes	χ^2^= 23.17	(<0.0001)	-1.06	(0.0003)	χ^2^= 0.98	(0.32)	N/A		χ^2^= 0.05	(0.83)	N/A	
New or change in medication (ref=no change)	AT1 receptor blocker					χ^2^=70.01	(<0.0001)	-0.64	(<0.0001)	χ^2^= 17.93	(0.02)	-0.59	(0.01)
	ACE inhibitor							-0.39	(0.003)			-0.24 (all others)	(0.25)
	Ca channel blocker							0.03	(0.88)				
	Beta blocker							-0.34	(0.14)				
	Diuretics							-0.48	(0.08)				
	Other single							0.07	(0.85)				
	Combination of 2 drugs							-0.48	(<0.0001)				
	Combination of 3 drugs							-0.68	(<0.0001)				
**GP**													
Sex (ref=male)	female	χ^2^= 1.23	(0.27)	N/A		χ^2^=1.84	(0.17)	N/A		χ^2^= 0.36	(0.55)	N/A	
Age		-0.01	(0.08)	collinear with years grad.		-0.02	(0.6)	collinear with years grad.		-0.001	(0.85)	collinear with years grad.	
Years graduation		-0.01	(0.05)	0.002	(0.75)	-0.06	(0.07)	0.004	(0.33)	0.004	(0.55)	N/A	
Place graduation (ref=Australia)	overseas	χ^2^= 0.89	(0.35)	N/A		χ^2^=5.89	(0.01)	-0.06	(0.54)	χ^2^=1.47	(0.23)	N/A	
**Clinic**													
Size		-0.01	(0.45)	N/A		-0.02	(0.32)	N/A		-0.02	(0.25)	N/A	
Location (ref=Metropolitan)	rural	χ^2^= 0.14	(0.71)	N/A		χ^2^= 6.20	(0.01)	0.04	(0.72)	χ^2^= 0.05	(0.82)	N/A	

*Only clinics with 20 or more patients were included in the analyses.

**Only clinics with 20 or more patients and patients with at least 1 BP measure were included in the analyses.

†Chi square test for categorical variables and logistic regression estimate for continuous variables.

Definitions: SHT=Severe hypertension; CHOL/HDL=Total cholesterol to high-density lipids ratio; AT1=Angiotensin 1; ACE=Angiotensin convertingenzyme; Ca=Calcium.

A hierarchical multivariate logistic regression model showed clustering by GP clinic after adjusting for patient characteristics (ICC = 10%, MOR = 1.8). Patient characteristics explained some of the model variance (Entropy R^2^ = 6.6%) while both GP and GP clinic characteristics had no influence.

### Variance in the achievement of normotension

The median and standard deviation in the percentage of patients achieving normotension among the 106 GP clinics with at least 20 SHT patients were 39% and 14% respectively (highest percentage of patients achieving normotension was 75%, lowest rate 0%) (see Figure 3). There were 11 clinics for which no patient sustained normotension (see Figure 3), and the difference in normotension rates across GP clinics was statistically significant (p_*Fisher*_ < 0.0001).

Clinics with higher rates of follow-up had, on average, higher rates of achievement of normotension (p < 0.005). Rates of sustained normotension were low and did not show an association with follow-up rates.

Male patients and patients with unknown CHOL:HDL were independently less likely to achieve normotension, but not less likely to sustain it. The issue of a prescription of some drugs during the year of follow-up was independently associated with normotension. Patients prescribed AT-1 receptor blockers, ACE inhibitors and combinations of anti-hypertensive drugs were more likely to achieve normotension during the year of follow-up. In particular, prescription of AT-1 receptor blockers was also associated with sustaining normotension [odds ratio = 1.8, 95% CI = (1.1, 2.9)]. GP clinic location and GP country of graduation had an independent statistically significant association with achieving (but not sustaining) normotension, with clinics in metropolitan areas and GPs graduated in Australia performing slightly better (see Table 3).

When modelling BP control using a hierarchical multivariate regression model, none of the available GP and GP clinic characteristics had an effect. Both achieving and sustaining normotension were found to cluster by GP clinic after adjusting for patient characteristics (ICC = 6%, MOR = 1.5%).

## Discussion

This study was designed to explore responses by Australian GPs to SHT by looking at 4 basic measures: follow-up visits, initiation of, or changes to, antihypertensive drug treatment, and BP control. This is the first analysis that explores the variance in the response of GPs to measures of SHT.

Follow-up (94%) occurred with a median of 2 weeks, but only 8% of patients were referred to an appropriate specialist (at a median of 89 days). The follow-up time is better than that found in other studies [22], but only 2% were referred within 1 week, which is lower than the 4% compliance for this indicator found in the CareTrack Australia study [14]. Of the 6% of patients who had no follow-up, 93% had not had a measure of SHT in the past and were more likely to be perceived as being of low risk of hypertension-related disease. We also found that 14% of patients did not receive an initiation of, or change to, anti-hypertensive drug treatment, even though 63% of those for whom BP measures were available did not achieve normotension (versus 49% among those who had a change in medication). This is also consistent with previous work [22]. Overall, 5% of all patients (6% of those followed and with available BP measures) achieved and sustained normotension for the year of follow-up. Not all visits had an electronic record of blood pressure measurement. Conservatively assuming that BP in all the GP visits for which it was not recorded was in the normal range, no more than 7% of the followed patients would have achieved normotension and would have sustained it till the end of the study year.

The demographics of the GPs and GP clinics had very little effect on the observed management of SHT, and only some of the observed variance was explained by patient characteristics. Nevertheless, it is clear that some clinics performed better than others (see Figure 3).

### Limitations

This study provides a description of GP responses to the management of SHT and does not attempt to address the more complex issue of adherence to hypertension guidelines. All the patients’ information was extracted from the electronic records of the GPRN dataset. Therefore, visits by the same patient to other GP clinics or acute care centres were not accessed. It would have been desirable to include more information about patient comorbidities, as well as other patient outcomes such as stroke or heart attack rates; however this was considered beyond the scope of this study. There was no electronic record of a blood pressure measurement for 53% of the follow-up visits, a proportion of these missing measurements may have been recorded as free text, which was not available for this study. In this analysis, blood pressure has been treated as a categorical variable. Therefore, only changes in blood pressure across predefined categories were taken into account.

### Implications for policy, practice and further work

Decisions about care during a healthcare encounter are the result of a complex interplay among the demographics and co-morbidities of individual patients, the attitudes, knowledge and beliefs of both doctors and patients, and other socio-economic and clinical factors [28]. Nevertheless, this study suggests that severely hypertensive patients are not being managed aggressively enough, that more intensive treatments lead to better outcomes, and that it is possible for some practices to do better.

One possible explanation is the failing to escalate care in the presence of competing demands from multiple comorbidities [29,30]. For some patients, multidrug therapy or significant secondary effects from drugs may limit anti-hypertension treatment. For example, using AT1 receptor blockers and ACE inhibitors in elderly patients with renal impairment is not recommended, since they can worsen renal failure. Promoting and facilitating adherence to drug treatment (for example using dose administration aids) can improve BP control [31].

Also, the high prevalence of blood pressure above the normal range may make practitioners and patients more accepting of ongoing recording of high blood pressure. Poor understanding of the risks of SHT among some patients (such as younger males with unknown lipid measures and infrequent visits) could also be behind inappropriate follow-up and poor achievement of BP control. Absence of lipid measures might also reflect GP practices likely to be associated with failure to adequately follow up on hypertensive patients.

Adherence to simple processes of escalating care as necessary, in line with existing guidelines, would result in safer, more effective treatment [32]. Improved, more relevant and user-friendly tools for management of chronic conditions at the point of care are also needed. Recommendations have recently been made for national agreement on clinical standards for the basic care of common conditions, for developing tools to guide and document management, and for providing feedback to patients and practitioners. Developing and implementing these with respect to hypertension must be high on the national agenda, as it is clear that more needs to be done to improve the management of this dangerous condition.

## Conclusions

This study provides evidence of responses by Australian GP’s to measures of SHT using electronic health records. A large proportion of patients (94%) had at least one follow-up visit and initiation of, or changes to, antihypertensive drug treatment took place in 72% of patients. Nevertheless, blood pressure control in these patients was poor. It appears that more aggressive management of severe hypertension is needed and that it is possible for some practices to do better.

## Abbreviations

ACE: Angiotensin converting enzyme; AT1: Angiotensin 1; BP: Blood pressure; CHOL:HDL: Total cholesterol to high density lipoprotein ratio; GP: General practitioners; GPRN: General Practice Research Network; ICC: Intraclass correlation; MOR: Median value of the odds ratios’ second level in hierarchical model; SHT: Severe hypertension.

## Competing interests

The authors declare that they have no competing interests.

## Authors’ contributions

BG led the study design, data analysis and writing of the manuscript. BWR made substantial contributions to the study design, clinical interpretation of the findings and drafting of the manuscript. OPC contributed to the data analysis and writing of the manuscript. STL and RD participated in the interpretation of data and the writing. AD contributed to the data visualisation. EC participated in the design and coordination of the study. All authors have read and approved the final manuscript.

## Pre-publication history

The pre-publication history for this paper can be accessed here:

http://www.biomedcentral.com/1472-6963/13/414/prepub

## References

[B1] Kearney PM Whelton M Reynolds K Muntner P Whelton PK He J Global burden of hypertension: analysis of worldwide data Lancet 2005 365 9455 217 223 15652604 10.1016/S0140-6736(05)17741-1

[B2] WHO The Global Burden of Disease: 2004 Update 2008 Geneva, Switzerland: World Health Organization Press

[B3] Begg S Vos T Barker B Stevenson C Stanley L Lopez A The burden of disease and injury in Australia 2003 2007 Canberra: AIHW

[B4] Chobanian AV Bakris GL Black HR Cushman WC Green LA Izzo JL Jones DW Materson BJ Oparil S Wright JT Seventh report of the joint national committee on prevention, detection, evaluation, and treatment of high blood pressure Hypertension 2003 42 6 1206 1252 10.1161/01.HYP.0000107251.49515.c2 14656957

[B5] Collaboration PS Age-specific relevance of usual blood pressure to vascular mortality: a meta-analysis of individual data for one million adults in 61 prospective studies Lancet 2002 360 9349 1903 1913 12493255 10.1016/s0140-6736(02)11911-8

[B6] Levy D Larson MG Vasan RS Kannel WB Ho KKL The progression from hypertension to congestive heart failure JAMA 1996 275 1557 1562 10.1001/jama.1996.03530440037034 8622246

[B7] Sipahi I Tuzcu EM Schoenhagen P Wolski KE Nicholls SJ Balog C Crowe TD Nissen SE Effects of normal, pre-hypertensive, and hypertensive blood pressure levels on progression of coronary atherosclerosis J Am Coll Cardiol 2006 48 4 833 838 10.1016/j.jacc.2006.05.045 16904557

[B8] Milchak JL Carter BL James PA Ardery G Measuring adherence to practice guidelines for the management of hypertension Hypertension 2004 44 5 602 608 10.1161/01.HYP.0000144100.29945.5e 15381676 PMC1414061

[B9] McGlynn E Asch S Adams J Keesey J Hicks J DeCritofaro A Kerr E The quality of health care delivered to adults in the United States N Engl J Med 2003 348 2635 2645 10.1056/NEJMsa022615 12826639

[B10] Ardery G Carter BL Milchak JL Bergus GR Dawson JD James PA Franciscus C Kim Y Explicit and implicit evaluation of physician adherence to hypertension guidelines J Clin Hypertens 2007 9 2 113 119 10.1111/j.1524-6175.2007.06112.x PMC8110017 17268216

[B11] Heeley E Peiris D Patel A Cass A Weekes A Morgan C Anderson C Chalmers J Cardiovascular risk perception and evidence-practice gaps in Australian general practice (the AusHEART study) MJA 2010 195 5 254 259 20201758 10.5694/j.1326-5377.2010.tb03502.x

[B12] Barr ELM Zimmet PZ Welborn TA Jolley D Magliano DJ Dunstan DW Cameron AJ Dwyer T Taylor HR Tonkin AM Risk of cardiovascular and all-cause mortality in individuals with diabetes mellitus, impaired fasting glucose, and impaired glucose tolerance Circulation 2007 116 2 151 157 10.1161/CIRCULATIONAHA.106.685628 17576864

[B13] Carrington M Jain A Stewart S Pressure Points in Primary Care: A Study of Blood Pressure in 532,050 Patients in Australia from 2005 to 2010 2012 Melbourne, Australia: Baker IDI Health and Diabetes Institute

[B14] Runciman W Hunt T Hannaford N Hibbert P Westbrook J Coeira E Day R Hindmarsh D McGynn E Braithwaite J CareTrack: assessing the appropriateness of health care delivery in Australia Medical Journal of Australia 2012 197 2 100 105 10.5694/mja12.10510 22794056

[B15] Kamerow D Is hypertension really a neglected disease? BMJ 2010 340 c1353 10.1136/bmj.c1353

[B16] Oliveria SA Lapuerta P McCarthy BD L'Italien GJ Berlowitz DR Asch SM Physician-related barriers to the effective management of uncontrolled hypertension Arch Intern Med 2002 162 4 413 420 10.1001/archinte.162.4.413 11863473

[B17] Milchak JL Carter BL Ardery G Dawson JD Harmston M Franciscus CL Physician Adherence to Blood Pressure Guidelines and Its Effect on Seniors Pharmacotherapy 2008 28 7 843 851 10.1592/phco.28.7.843 18576899

[B18] Hyman DJ Pavlik VN Self-reported hypertension treatment practices among primary care physicians: blood pressure thresholds, drug choices, and the role of guidelines and evidence-based medicine Arch Intern Med 2000 160 15 2281 2286 10.1001/archinte.160.15.2281 10927724

[B19] NHF National Heart Foundation of Australia (National Blood Pressure and Vascular Disease Advisory Committee) Guide to Management of Hypertension 2008 2010

[B20] Marquez-Contreras E Coca A von Wichmann M D l F Divison JA Llisterri JL Sobrino J Filozof C Sanchez-Zamorano MA Grigorian Shamagian L Cardiovascular risk profile of uncontrolled hypertensive patients. The control-project study Med Clin 2007 128 3 86 91 10.1016/S0025-7753(07)72498-3 17288921

[B21] Preston RA Baltodano NM Cienki J Materson BJ Clinical presentation and management of patients with uncontrolled, severe hypertension: results from a public teaching hospital J Hum Hypertens 1999 13 4 249 255 10.1038/sj.jhh.1000796 10333343

[B22] Borzecki AM Kader B Berlowitz DR The epidemiology and management of severe hypertension J Hum Hypertens 2009 24 1 9 18 19440209 10.1038/jhh.2009.37 PMC3011090

[B23] Geoffrey PS Kevin M Andrew K Alice B Fiona H Leigh H Lisa S Sharon S The General Practice Research Network: the capabilities of an electronic patient management system for longitudinal patient data Pharmacoepidemiol Drug Saf 2003 12 483 489 10.1002/pds.834 14513662

[B24] Leyland A Goldstein H Multilevel Modelling of Health Statistics 2001 New York: Wiley

[B25] SAS Chapter 38: The Glimmix Procedure SAS/STAT 92 User's Guide 2008 Second North Carolina, USA: SAS Institute Inc. Last update 2010

[B26] Snijders TAB Bosker RJ Multilevel analysis:an introduction to basic and advanced multilevel modeling 1999 London ESIY 1SP: SAGE Publications Ltd

[B27] Larsen K Merlo J Appropriate assessment of neighborhood effects on individual health: integrating random and fixed effects in multilevel logistic regression Am J Epidemiol 2005 161 1 81 88 10.1093/aje/kwi017 15615918

[B28] Kennedy P Leathley C Hughes C Clinical practice variation MJA 2010 193 8 S97 S99 20955142 10.5694/j.1326-5377.2010.tb04021.x

[B29] Stange KC Is 'Clinical Inertia’ blaming without understanding? Are competing demands excuses? Annals Fam Med 2007 5 4 371 374 10.1370/afm.734

[B30] van Bruggen R Gorter K Stolk R Klungel O Rutten G Clinical inertia in general practice: widespread and related to the outcome of diabetes care Fam Pract 2009 26 6 428 436 10.1093/fampra/cmp053 19729401

[B31] Connor J Rafter N Rodgers A Do fixed-dose combination pills or unit-of-use packaging improve adherence? A systematic review Bull World Health Organ 2004 82 12 935 939 15654408 PMC2623099

[B32] Do Guidelines Make a Difference in Patienthttp://www.nhmrc.gov.au/nics/materials-and-resources/do-guidelines-make-difference-health-outcomes

